# Integrating Meaning and Purpose: A Spiritually-Informed Approach to Psychiatric Care

**DOI:** 10.7759/cureus.90567

**Published:** 2025-08-20

**Authors:** Ryan V Akhavan, Dan Erdman, Mirbelys Perera

**Affiliations:** 1 Surgery, St. George's University School of Medicine, St. George's, GRD; 2 Clinical Research, St. George's University School of Medicine, St. George's, GRD; 3 Psychiatry, Keralty Community Hospital, Miami, USA

**Keywords:** bahá'í, case report, depression, meaning, psychiatric care, purpose, spiritual psychotherapy, virtues

## Abstract

Depression and suicidality are typically treated with a combination of medication and cognitive-behavioral therapy. However, for many individuals, especially those dealing with trauma or a loss of meaning, these approaches may not fully address the deeper layers of distress. Emerging research points to the potential value of incorporating spiritual and meaning-centered practices into psychiatric care. This case report describes a 24-year-old mixed-race woman who presented with suicidality following a sexual assault and long-standing feelings of purposelessness. After stabilization, she was referred to spiritually integrated therapy by her outpatient psychiatrist and also received support from her spiritual community. Her treatment centered on developing spiritual qualities, building a sense of purpose, and engaging in acts of service. Over time, she showed significant emotional and psychological improvement. Her case highlights how meaning-focused and spiritually informed care can support long-term healing, especially in individuals facing existential challenges.

## Introduction

The search for meaning, identity, and purpose is central to psychological health. Although spirituality and healing were once deeply intertwined, from indigenous rituals to early philosophical traditions, modern psychiatry often sidelines these dimensions in favor of diagnostic labels and symptom management. Yet growing research supports the value of spiritually integrated psychotherapy (SIP) in addressing existential suffering. For instance, a survey of patients with major depression found high interest in integrating spirituality into mental healthcare [1]. Moreover, randomized trials of spiritually oriented interventions demonstrate significant reductions in depressive symptoms and suicidal ideation for religious clients [2].

Logotherapy, originated by Viktor Frankl and grounded in meaning-centered practice, is a therapeutic approach that emphasizes finding meaning in life, including through spiritual exploration, as a pathway to psychological well-being. While spirituality has been increasingly recognized in mental health research, the specific dimensions of meaning and purpose remain under-documented in the literature. Logotherapy addresses this gap by offering structured methods to explore these dimensions, and it also shows substantial clinical benefits. In a Taiwanese study, patients receiving 12 weeks of logotherapy experienced meaningful gains in life purpose and significant reductions in depression, hopelessness, and suicidal ideation [3]. Group logotherapy has also been shown to enhance meaning in life and reduce depressive symptoms among university students [4]. Clinicians like Kenneth Pargament and Viktor Frankl provide ethical and theoretical foundations for spiritually integrated, values-centered therapy while cautioning against imposing belief systems [5].

From a Bahá’í informed perspective, as detailed by Danesh in The Psychology of Spirituality, humans are viewed as souls in development, called to cultivate virtues, face challenges, and contribute to society [6]. When therapy encourages the exploration of compassion, resilience, truthfulness, and service, it can offer more profound healing than symptom management alone.

This report examines the case of a young woman whose recovery was enhanced through a spiritual and meaning-centered framework, resulting in measurable improvements in both her mental health and her quality of life.

## Case presentation

A 24-year-old mixed-race female college student was brought to the emergency department after expressing suicidal ideation and writing a farewell note. She reported persistent feelings of worthlessness, disconnection, and despair. On psychiatric evaluation, she disclosed a history of sexual assault at age 23, after which she experienced chronic symptoms of post-traumatic stress, including nightmares, hypervigilance, emotional numbing, and avoidance behaviors. Her academic performance declined, and she increasingly isolated herself from peers and family.

She described feeling “empty,” lacking direction, and frequently asked herself: “Why am I even here?” or “What’s the point of my life?” A standardized pre- and post-intervention assessment of depression and suicidal ideation was not retrievable due to difficulties in long-term outpatient follow-up, though symptom changes were tracked through the patient health questionnaire (PHQ)-9 and clinician progress notes. She reported frequent crying spells, disrupted sleep, a diminished appetite, and passive thoughts of death. She denied current suicidal intent or plan but acknowledged that these thoughts had become more frequent and distressing. She was not on any psychiatric medication and had no prior formal therapy. Her family, while supportive, had limited awareness of her internal experience.

Mental status exam

The patient was alert and oriented to person, place, time, and situation. Appearance was consistent with stated age, with fair grooming and hygiene. Speech was soft but coherent, with a normal rate and rhythm. Mood was described as “depressed,” with a congruent, constricted affect. Thought processes were logical and goal-directed, without evidence of formal thought disorder. Thought content was notable for passive suicidal ideation without plan or intent, and no homicidal ideation. No perceptual disturbances were reported or observed. Insight and judgment were fair, and attention and concentration were intact during the interview. Memory was grossly preserved.

The patient was admitted to inpatient psychiatry and initiated on fluoxetine for depressive symptoms and prazosin for nightmares. Upon stabilization, she was discharged with outpatient follow-up. As part of her discharge planning, she was counseled to pursue spiritually integrated psychotherapy both through an outpatient psychiatrist and with a trusted mentor from her spiritual community, who followed a structured support protocol. This protocol consisted of weekly one-on-one discussions and guided journaling prompts, including: “What do I want to be remembered for?”, “What qualities do I admire most in others?” and “How can I use what I’ve been through to help others?” She agreed to this approach and was referred accordingly.

Her outpatient treatment included six guided reflection sessions on themes such as identity beyond trauma, the human capacity to develop spiritual virtues, and the transformative power of service. The patient resonated deeply with the prompts and discussions, stating that it was the first time she had considered that her life could still have meaning beyond what had happened to her.

Over time, she began to find solace and comfort in believing that a higher power was present in her life, that she was not alone in her suffering. The realization that she had been created for a purpose and that someone greater than herself was watching over her became a decisive protective factor. This spiritual connection helped her feel supported during moments of distress and renewed her sense of strength and meaning in her day-to-day life.

She was introduced to community service opportunities, including a youth mentorship program, and began participating in weekly group reflection circles focused on themes of justice, compassion, and truthfulness. Over the course of eight weeks, the patient showed steady improvement, as measured through serial PHQ-9 assessments and clinician-rated progress notes. Her sleep stabilized, her appetite returned, and she became more emotionally expressive. Her PHQ-9 score dropped from 21 (severe) to 9 (mild). She verbalized a renewed sense of purpose and reported fewer intrusive memories. She was maintained on fluoxetine and continued in spiritually integrated therapy with her outpatient psychiatrist.

## Discussion

This case highlights how meaning-centered and spiritually integrated care can serve as a powerful adjunct to traditional psychiatric treatment, particularly in trauma-affected populations. A growing body of literature supports the inclusion of spirituality in mental health care. In a meta-analysis by Captari et al., spiritually integrated interventions were found to be significantly more effective than control conditions in reducing symptoms of depression, anxiety, and post traumatic stress disorder (PTSD) [7].

Research also shows that patients who explore their spiritual beliefs and develop a sense of meaning and purpose tend to demonstrate greater resilience and stronger engagement with therapy [8]. Logotherapy, developed by Viktor Frankl, emphasizes that finding meaning in suffering can be a key driver of healing in traumatized individuals [3]. Other studies suggest that a strong sense of spiritual identity and alignment with personal values may buffer against suicidality and psychological collapse [9].

Clinicians must approach spiritual work with cultural humility and ethical sensitivity. Spiritually integrated psychotherapy is not about promoting a particular worldview but allowing patients to explore their values, beliefs, and life narratives in a supportive setting [10]. From a Bahá’í perspective, a religion that embraces the unity of all faiths and belief systems, as articulated in The Psychology of Spirituality by Danesh, the purpose of life is to grow spiritually, serve others, and cultivate qualities such as love, courage, and integrity [6]. Within this framework, suffering can be reframed not as meaningless pain but as an opportunity for transformation and connection.

In this case, the patient experienced not only a reduction in depressive and post-traumatic symptoms but also a meaningful shift in self-concept, from a traumatized victim to a resilient young woman with a renewed sense of purpose. Her recovery was strengthened by her engagement in service, reflection, and spiritual exploration through her outpatient care and community support, allowing her to reclaim agency and direction in her life.

## Conclusions

Incorporating spiritually integrated psychotherapy into psychiatric care can offer meaningful improvements in both symptoms and quality of life. For patients facing existential distress, especially following trauma, the exploration of meaning, purpose, and spiritual identity may be essential for long-term healing.

This case also underscores the vital role of community in the recovery process. Supportive networks, whether through faith-based groups, mentorship, or organized service activities, can help patients feel less isolated, reinforce protective factors, and provide practical opportunities to apply therapeutic insights in daily life. The integration of community involvement with spiritual exploration offers a more holistic approach to psychiatric care, one that not only addresses symptoms but also strengthens resilience, fosters belonging, and sustains personal growth beyond the clinical setting.

This case adds to the growing evidence that purpose-based, virtue-centered approaches, supported by meaningful community engagement, are ethically appropriate and clinically effective.
